# Supplementary material for: Transcriptome Analysis for Genes Associated with Small Ruminant Lentiviruses Infection in Goats of Carpathian Breed
Source: Viruses. 2021 Oct 13;13(10):2054. doi: 10.3390/v13102054 (PMC8538975; doi:10.3390/v13102054)
Supplement: Supplementary file 1 [file viruses-13-02054-s001.zip › Supplementary file 1 Figure S1.pdf]

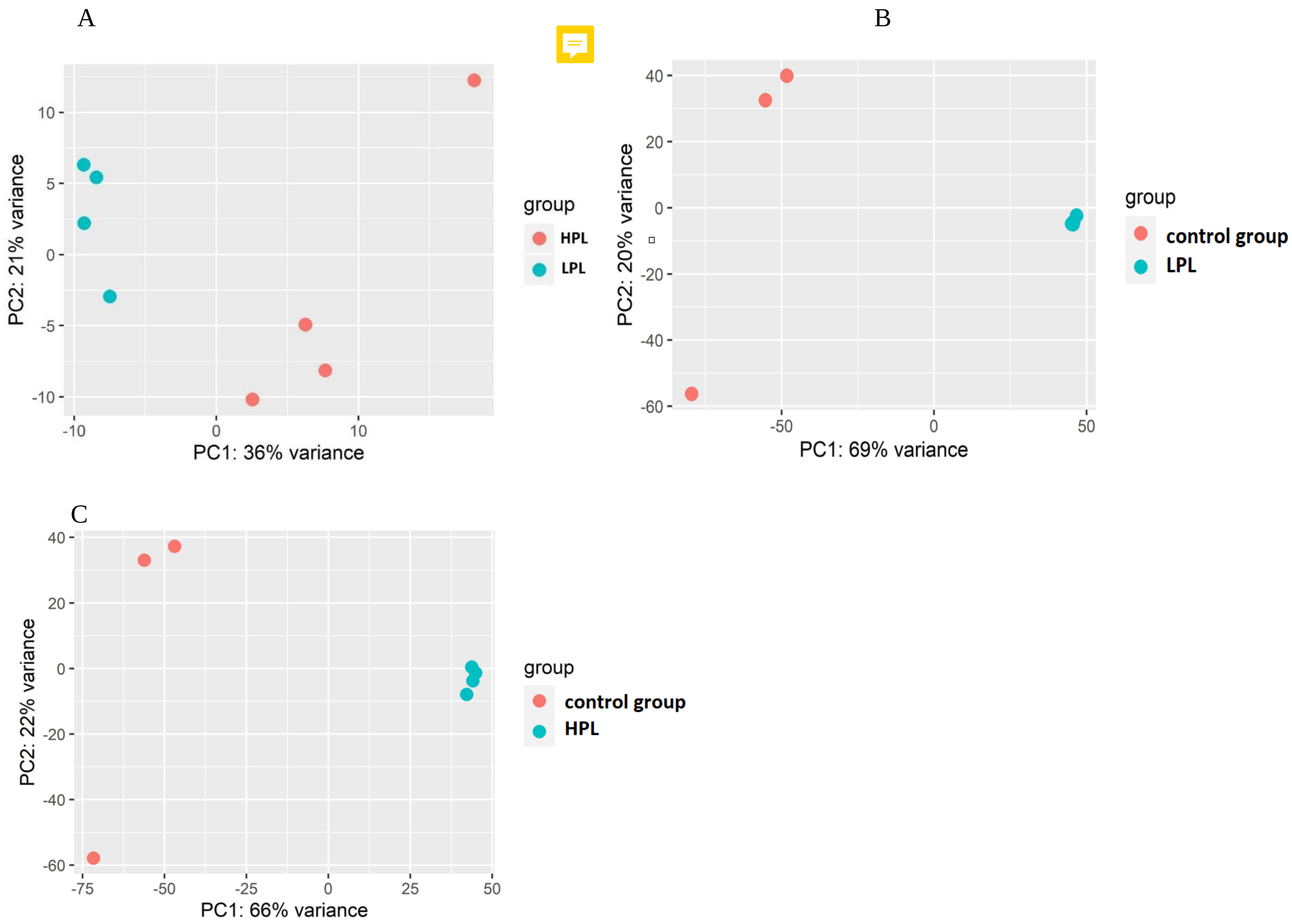

Figure S1. Principal component analysis (PCA) plot presented samples clustering based on similarities of their gene expression variation. A - goats with high (HPL) and low (LPL) proviral load are shown as red and blue circles, respectively; B - goats represented control group and low (LPL) proviral load are shown as red and blue circles, respectively; C - goats represented control group and low (LPL) proviral load are shown as red and blue circles, respectively;
