# Supplementary material for: Transcriptome Analysis for Genes Associated with Small Ruminant Lentiviruses Infection in Goats of Carpathian Breed
Source: Viruses. 2021 Oct 13;13(10):2054. doi: 10.3390/v13102054 (PMC8538975; doi:10.3390/v13102054)
Supplement: Supplementary file 1 [file viruses-13-02054-s001.zip › Supplementary file 1 Table S1.pdf]

Table S1.The primers used for real-time PCR method.

Gene symbol	Accession number	Primers	Amplicon lenght (bp)
CCL2	ENSCHIG00000023567	F ATGAAGGTCTCCGCTGCTCT<br>R TGCTGGTGGTGACTCTTCTG	165
IL15	ENSCHIG00000024482	F AGAGGCTGGCATTCATGTCT<br>R GCTCAATTGTTTCCAAATCATGT	102
CXCR3	ENSCHIG00000014964	F TGAGCAGCACTGACACCTTT<br>R GCCCCTGCATAGAAGTTGAT	163
MIF	ENSCHIG00000022871	F GTGGTCCCAGACCAGCTCAT<br>R CGTTCATGTCGCAGAAGTTG	192
NOD2 v3	ENSCHIG00000024746	F TCCTGAGCACCTATGATGGAG<br>R TTAAAATGGTCACGGGTGCT	160
BCL2	ENSCHIG00000016322	F GCAGCTCTAACTGGAGAGTTCTG<br>R TTCTGGTGTTTCCCTCTTGG	230
ITK	ENSCHIG00000027054	F AACCAGCTTGGCGTACTTTG<br>R TGACATGGGATGATAATGTCACT	121
CCR	ENSCHIG00000020624	F CTTTGGTTTTGTGGGCAACT<br>R TGGTCTGCAGCATAGTGAGC	153
CXCL5	ENSCHIG00000010442	F GAGCTGCGTTGTGTGTGTTT<br>R ACTTCCACCTTGGAGCACTG	104
HPRT	ENSCHIG00000015745	F TGGCTCGAGATGTGATGAAG<br>R TATGTCGCCTGTTGACTGGT	197
