# Supplementary material for: Transcriptome Analysis for Genes Associated with Small Ruminant Lentiviruses Infection in Goats of Carpathian Breed
Source: Viruses. 2021 Oct 13;13(10):2054. doi: 10.3390/v13102054 (PMC8538975; doi:10.3390/v13102054)
Supplement: Supplementary file 1 [file viruses-13-02054-s001.zip › Supplementary file 1 Table S2.pdf]

Table S2. Summary of reads quality and mapping results of RNA-seq.

Sample	Proviral load (copies/500 ng of DNA)	Number of filtered reads	Number of reads mapped	Percent of mapping (%)	Number of reads mapped to annotation database	Percent of reads mapped to annotation database
15	13	25879879	21945307	84.8	11010428	50.2
25	16	34062835	32020903	94.0	15762381	49.2
16	23	32626721	30990435	95.0	14047550	45.3
30	14	37205971	35353941	95.0	16741542	47.4
31	97	36269260	34930596	96.3	17889765	51.2
32	80	34154210	32608846	95.5	16660908	51.1
33	79	33127821	31039572	93.7	15785337	50.9
8	73	40923520	38990584	95.3	18592969	47.7
44	0	30026684	18316277	61.0	14084851	46.9
45	0	16510450	10401583	63.1	5818169	35.2
46	0	13837931	6780586	49.0	3983817	28.8
