# Supplementary material for: Transcriptome Analysis for Genes Associated with Small Ruminant Lentiviruses Infection in Goats of Carpathian Breed
Source: Viruses. 2021 Oct 13;13(10):2054. doi: 10.3390/v13102054 (PMC8538975; doi:10.3390/v13102054)
Supplement: Supplementary file 1 [file viruses-13-02054-s001.zip › Supplementary file 1 Table S3.pdf]

Table S3. The most significantly dysregulated genes between HPL and LPL goats

Gene symbol	Accession number	Gene name	FC
up-regulated in HPL goats			
DPYS	ENSCHIG00000025287	Dihydropyrimidinase	5.94
ZNF697	ENSCHIG00000014100	Zinc Finger Protein 697	4.87
GPRIN3	ENSCHIG00000008090	G protein-regulated inducer of neurite outgrowth 3	4.23
OR52L1	ENSCHIG00000007356	Olfactory receptor family 52 subfamily L member 1	4.14
EHD4	ENSCHIG00000025291	EH domain-containing protein 4	4.01
OCLN	ENSCHIG00000001852	Occludin	3.79
SULT6B1	ENSCHIG00000024550	Sulfotransferase 6B1	3.71
TRPM5	ENSCHIG00000002319	Transient receptor potential cation channel subfamily M member 5	3.65
IFITM1	ENSCHIG00000019798	Interferon induced transmembrane protein 1	3.62
DRAM1	ENSCHIG00000019601	DNA damage-regulated autophagy modulator protein 1	3.60
down-regulated in HPL goats			
SLC22A1	ENSCHIG00000014819	Solute carrier family 22 member 1	-419.33
FTL	ENSCHIG00000011303	Ferritin light chain	-190.61
HBA1	ENSCHIG00000024771	Hemoglobin subunit alpha 1	-166.75
HBAII	ENSCHIG00000019220	Hemoglobin subunit alpha-2-like	-155.25
BTF3	ENSCHIG00000008661	Basic transcription factor 3	-129.90
ALAS2	ENSCHIG00000011854	5'-aminolevulinate synthase 2	-110.62
B2M	ENSCHIG00000018922	Beta-2-microglobulin	-96.08
MAL	ENSCHIG00000020112	Malate dehydrogenase	-79.11
HBB	ENSCHIG00000017796	Hemoglobin subunit beta	-62.07
LGALS9	ENSCHIG00000019679	Galectin-9	-57.83

HPL- high proviral load
LPL- low proviral load
