# Supplementary material for: Transcriptome Analysis for Genes Associated with Small Ruminant Lentiviruses Infection in Goats of Carpathian Breed
Source: Viruses. 2021 Oct 13;13(10):2054. doi: 10.3390/v13102054 (PMC8538975; doi:10.3390/v13102054)
Supplement: Supplementary file 1 [file viruses-13-02054-s001.zip › Supplementary file 1 Table S5.pdf]

Table S5. The detail fold change values and GO term analysis of upregulated genes in group HPL goats.

GO	Number of genes	Genes	Name of genes	FC
**peptidyl-cysteine modification**	**7**	*GOLGA7B*	golgin A7 family member B	4.96
		MAP6D1	MAP6 domain containing 1	2.99
		TMX3	thioredoxin related transmembrane protein 3	2.36
		RAB3D	RAB3D, member RAS oncogene family	2.33
		ZDHHC23	zinc finger DHHC-type containing 23	2.24
		RAB6A	RAB6A, member RAS oncogene family	2.11
		ZDHHC22	zinc finger DHHC-type containing 22	2.01
**cytokine production**	**95**	*ADAMTS3*	ADAM metallopeptidase with thrombospondin type 1 motif 3	3.00
		IL15	interleukin 15	2.77
		CMKLR1	chemerin chemokine-like receptor 1	2.61
		NOD2	nucleotide binding oligomerization domain containing 2	2.55
		ABCA1	ATP binding cassette subfamily A member 1	2.48
		GDF2	growth differentiation factor 2	2.44
		CCR2	C-C motif chemokine receptor 2	2.44
		HFE	homeostatic iron regulator	2.37
		RSAD2	radical S-adenosyl methionine domain containing 2	2.36
		CHUK	conserved helix-loop-helix ubiquitous kinase	2.33
		ITK	IL2 inducible T cell kinase	2.27
		LBP	lipopolysaccharide binding protein	2.24
		ADCY7	adenylate cyclase 7	2.17
		IL6R	interleukin 6 receptor	2.16
		IL1A	interleukin 1 alpha	2.16
		RAB7B	RAB7B, member RAS oncogene family	2.15
		IFIH1	interferon induced with helicase C domain 1	2.14
		C3AR1	complement C3a receptor 1	2.00
		LPL	lipoprotein lipase	1.96
		CD274	CD274 molecule	1.96
**vacuole organization**	**34**	*ABCA1*	ATP binding cassette subfamily A member 1	2.48
		PIP4K2B	phosphatidylinositol-5-phosphate 4-kinase type 2 beta	2.46
		UBXN2A	UBX domain protein 2A	2.28
		RALB	RAS like proto-oncogene B	2.27
		RAB7B	RAB7B, member RAS oncogene family	2.15
		PPT1	palmitoyl-protein thioesterase 1	2.06
		GAA	glucosidase alpha, acid	2.01
		ACP2	acid phosphatase 2, lysosomal	1.95
		TBC1D14	TBC1 domain family member 14	1.68
		AKTIP	AKT interacting protein	1.64
**vesicle organization**	**38**	*SEC16B*	SEC16 homolog B, endoplasmic reticulum export factor	6.08
		RAB8B	RAB8B, member RAS oncogene family	2.87
		ABCA1	ATP binding cassette subfamily A member 1	2.48
		SAMD9	sterile alpha motif domain containing 9	2.19
		VPS39	VPS39, HOPS complex subunit	2.17
		RAB7B	RAB7B, member RAS oncogene family	2.15
		DYSF	dysferlin	2.02
		STX19	syntaxin 19	2.01
		EXOC8	exocyst complex component 8	1.88
		BCL2	BCL2, apoptosis regulator	1.83
**glutamate receptor signaling pathway**	**5**	*CACNG3*	calcium voltage-gated channel auxiliary subunit gamma 3	4.21
		GRM2	glutamate metabotropic receptor 2	3.01
		CRHBP	corticotropin releasing hormone binding protein	2.37
		HOMER2	homer scaffold protein 2	1.87
		PRNP	prion protein	1.86
		MAB21L2	mab-21 like 2	6.44
		IHH	Indian hedgehog signaling molecule	5.02
		PAX9	paired box 9	3.34

		COL1A1	collagen type I alpha 1 chain	2.97
		DKK1	dickkopf WNT signaling pathway inhibitor 1	2.47
		SGPL1	sphingosine-1-phosphate lyase 1	1.99
**carbohydrate derivative transport**	**15**	*SLC28A2*	solute carrier family 28 member 2	6.09
		SLC17A3	solute carrier family 17 member 3	4.03
		ABCG1	ATP binding cassette subfamily G member 1	2.33
		SLC35A3	solute carrier family 35 member A3	2.15
		SLC29A3	solute carrier family 29 member 3	2.12
		GJA1	gap junction protein alpha 1	1.93
		SLC37A2	solute carrier family 37 member 2	1.74
		SLC35D1	solute carrier family 35 member D1	1.66
		SLC28A3	solute carrier family 28 member 3	1.62
		SLC35A5	solute carrier family 35 member A5	1.60
**respiratory gaseous exchange**	**5**	*SFTPB*	surfactant protein B	7.08
		EDN1	endothelin 1	3.76
		NDST1	N-deacetylase and N-sulfotransferase 1	2.05
		GAA	glucosidase alpha, acid	2.01
		MAFB	MAF bZIP transcription factor B	1.84
**tissue remodeling**	**14**	*IHH*	Indian hedgehog signaling molecule	5.02
		DCSTAMP	dendrocyte expressed seven transmembrane protein	4.97
		RASSF2	Ras association domain family member 2	2.92
		CCR2	C-C motif chemokine receptor 2	2.44
		DLL4	delta like canonical Notch ligand 4	2.43
		RAB3D	RAB3D, member RAS oncogene family	2.33
		IL1A	interleukin 1 alpha	2.16
		EFNA2	ephrin A2	2.14
		NOTCH2	notch receptor 2	2.04
		CLDN18	claudin 18	2.01
**dopamine receptor signaling pathway**	**3**	*NSG2*	neuronal vesicle trafficking associated 2	13.88
		NSG1	neuronal vesicle trafficking associated 1	4.19
		DRD1	dopamine receptor D1	2.81
