# Supplementary material for: Transcriptome Analysis for Genes Associated with Small Ruminant Lentiviruses Infection in Goats of Carpathian Breed
Source: Viruses. 2021 Oct 13;13(10):2054. doi: 10.3390/v13102054 (PMC8538975; doi:10.3390/v13102054)
Supplement: Supplementary file 1 [file viruses-13-02054-s001.zip › Supplementary file1 Table S4.pdf]

Table S4. The detail fold change values and GO term analysis of down-regulated genes in group HPL goats.

GO	Number of genes	Genes	Name of genes	FC
**execution phase of apoptosis**	**10**	*PTGIS*	prostaglandin I2 synthase	-40.28
		ENDOG	endonuclease G	-2.47
		BOK	BCL2 family apoptosis regulator BOK	-2.42
		ERN2	endoplasmic reticulum to nucleus signaling 2	-2.21
		SHARPIN	SHANK associated RH domain interactor	-2.20
		CIDEC	cell death inducing DFFA like effector c	-1.85
		CXCR3	C-X-C motif chemokine receptor 3	-1.76
		SIRT2	sirtuin 2	-1.58
		RPS3	ribosomal protein S3	-1.55
		BAX	BCL2 associated X, apoptosis regulator	-1.47
**tetrapyrrole metabolic process**	**9**	*ALAS2*	5'-aminolevulinate synthase 2	-109.21
		ALAD	aminolevulinate dehydratase	-2.36
		UROD	uroporphyrinogen decarboxylase	-2.27
		CYP1A2	cytochrome P450, family 1, subfamily A, polypeptide 2	-2.20
		UROS	uroporphyrinogen III synthase	-2.18
		HNF1A	HNF1 homeobox A	-1.76
		MMAB	metabolism of cobalamin associated B	-1.76
		ATP5IF1	ATP synthase inhibitory factor subunit 1	-1.74
		BLVRB	biliverdin reductase B	-1.62
**response to toxic substance**	43	*HBM*	hemoglobin subunit mu	-29.13
		CHRNB2	cholinergic receptor nicotinic beta 2 subunit	-4.83
		LYPD1	LY6/PLAUR domain containing 1	-4.83
		GNRH1	gonadotropin releasing hormone 1	-4.48
		MPO	myeloperoxidase	-3.74
		CHRNA6	cholinergic receptor nicotinic alpha 6 subunit	-3.68
		DRD3	dopamine receptor D3	-3.26
		CHRND	cholinergic receptor nicotinic delta subunit	-2.93
		LTC4S	leukotriene C4 synthase	-2.73
		IL6	interleukin 6	-2.49
**reactive nitrogen species metabolic process**	**5**	*PTGIS*	prostaglandin I2 synthase	-40.28
		PKD2	polycystin 2, transient receptor potential cation channel	-3.51
		NOS3	nitric oxide synthase 3	-2.27
		CPS1	carbamoyl-phosphate synthase 1	-2.27
		DDAH2	dimethylarginine dimethylaminohydrolase 2	-1.95
**regulation of signaling receptor activity**	**53**	*FGF16*	fibroblast growth factor 16	-8.29
		CCL2	chemokine (C-C motif) ligand 2	-7.83
		CGA	glycoprotein hormones, alpha polypeptide	-7.58
		INHBE	inhibin subunit beta E	-7.45
		CLEC11A	C-type lectin domain containing 11A	-5.36
		CXCL5	chemokine (C-X-C motif) ligand 5	-5.36
		TNFSF11	TNF superfamily member 11	-4.98
		AVP	arginine vasopressin	-4.83
		LYPD1	LY6/PLAUR domain containing 1	-4.83
		FOXH1	forkhead box H1	-4.70
		GNRH1	gonadotropin releasing hormone 1	-4.48
		NPY	neuropeptide Y	-3.87
		CCL17	C-C motif chemokine ligand 17	-3.83
		OXT	oxytocin/neurophysin I prepropeptide	-3.65
		GHRH	growth hormone releasing hormone	-3.55
		NOG	noggin	-3.29
		EDA	ectodysplasin A	-3.17
		CXCL9	C-X-C motif chemokine ligand 9	-3.04
		MIF	macrophage migration inhibitory factor	-3.03
		RETN	resistin	-2.73
**peptidyl-arginine modification**	**10**	*ART1*	ADP-ribosyltransferase 1	-2.69
		PADI6	peptidyl arginine deiminase 6	-2.30
		PADI3	peptidyl arginine deiminase 3	-2.08

		PADI1	peptidyl arginine deiminase 1	-2.05
		KRTCAP2	keratinocyte associated protein 2	-1.81
		COPRS	coordinator of PRMT5 and differentiation stimulator	-1.75
		PARK7	Parkinsonism associated deglycase	-1.55
		PRMT7	protein arginine methyltransferase 7	-1.54
		PRMT2	protein arginine methyltransferase 2	-1.48
		PRMT1	protein arginine methyltransferase 1	-1.46
**amine transport**	**15**	*CHRNB2*	cholinergic receptor nicotinic beta 2 subunit	-4.83
		CHRNA6	cholinergic receptor nicotinic alpha 6 subunit	-3.68
		DRD3	dopamine receptor D3	-3.26
		ACE2	angiotensin I converting enzyme 2	-2.55
		SNCG	synuclein gamma	-2.54
		SYT2	synaptotagmin 2	-2.30
		SLC22A16	solute carrier family 22 member 16	-2.27
		SYT1	synaptotagmin 1	-2.27
		SLC18A1	solute carrier family 18 member A1	-2.08
		DTNBP1	dystrobrevin binding protein 1	-1.79
**organic cation transport**	**3**	*SLC22A1*	solute carrier family 22 member 1	-427.65
		SLC22A16	solute carrier family 22 member 16	-2.27
		SLC18A3	solute carrier family 18 member A3	-2.23
**ammonium transport**	**15**	*RHAG*	Rh associated glycoprotein	-8.59
		CHRNB2	cholinergic receptor nicotinic beta 2 subunit	-4.83
		CHRNA6	cholinergic receptor nicotinic alpha 6 subunit	-3.68
		DRD3	dopamine receptor D3	-3.26
		SLC6A2	solute carrier family 6 member 2	-2.59
		SNCG	synuclein gamma	-2.54
		SYT2	synaptotagmin 2	-2.30
		ADCYAP1	adenylate cyclase activating polypeptide 1	-2.27
		SLC22A16	solute carrier family 22 member 16	-2.27
		SYT1	synaptotagmin 1	-2.27
**NADH dehydrogenase complex assembly**	**21**	*NDUFA13*	NADH:ubiquinone oxidoreductase subunit A13	-2.73
		NDUFS5	NADH:ubiquinone oxidoreductase subunit S5	-2.27
		NDUFS7	NADH:ubiquinone oxidoreductase core subunit S7	-2.13
		NDUFB9	NADH:ubiquinone oxidoreductase subunit B9	-1.96
		NDUFA9	NADH:ubiquinone oxidoreductase subunit A9	-1.94
		NDUFA8	NADH:ubiquinone oxidoreductase subunit A8	-1.76
		BCS1L	BCS1 homolog, ubiquinol-cytochrome c reductase complex chaperone	-1.76
		NDUFB2	NADH:ubiquinone oxidoreductase subunit B2	-1.66
		NDUFA5	NADH:ubiquinone oxidoreductase subunit A5	-1.55
		NDUFA2	NADH:ubiquinone oxidoreductase subunit A2	-1.51
